# Supplementary material for: Loss of Fmr1 reorganizes the multi-elemental composition across tissues in Fragile X Syndrome mice
Source: PLoS One. 2026 Jul 10;21(7):e0352693. doi: 10.1371/journal.pone.0352693 (PMC13354080; doi:10.1371/journal.pone.0352693)
Supplement: S1 File — Density overlays validating global model fit for representative elements (Mg, Na, and K). The solid black line y defines the empirical distribution of the observed log-ratio data; thin turquoise lines yrep represent 50 independent simulated datasets generated from the posterior predictive distribution. High alignment between empirical and simulated densities verifies structural model adequacy and distributional assumptions. (DOCX) [file pone.0352693.s001.docx]

**Figure S1.** Multivariate posterior predictive checks (PPC) for core elemental indicators. Density overlays validating global model fit for representative elements (Mg, Na, and K). The solid black line *y* defines the empirical distribution of the observed log-ratio data; thin turquoise lines *y*_rep_ represent 50 independent simulated datasets generated from the posterior predictive distribution. High alignment between empirical and simulated densities verifies structural model adequacy and distributional assumptions.
