# Supplementary material for: Loss of Fmr1 reorganizes the multi-elemental composition across tissues in Fragile X Syndrome mice
Source: PLoS One. 2026 Jul 10;21(7):e0352693. doi: 10.1371/journal.pone.0352693 (PMC13354080; doi:10.1371/journal.pone.0352693)
Supplement: S2 File — Standalone posterior histograms split by individual sampling chains for intercept parameters (Mg, Na, and K). Overlapping unimodal distributions across all four computational chains visually confirm robust parameter space exploration, chain mixing, and global convergence. (DOCX) [file pone.0352693.s002.docx]

**Figure S2.** Markov Chain Monte Carlo (MCMC) sampling diagnostics. Standalone posterior histograms split by individual sampling chains for intercept parameters (Mg, Na, and K). Overlapping unimodal distributions across all four computational chains visually confirm robust parameter space exploration, chain mixing, and global convergence.
