# Supplementary material for: Loss of Fmr1 reorganizes the multi-elemental composition across tissues in Fragile X Syndrome mice
Source: PLoS One. 2026 Jul 10;21(7):e0352693. doi: 10.1371/journal.pone.0352693 (PMC13354080; doi:10.1371/journal.pone.0352693)
Supplement: S3 File — Points represent median and lines represent 95% HPD. (DOCX) [file pone.0352693.s003.docx]

**Figure S3.** Forest plot displaying the median residual correlation coefficients and corresponding 95% HPD intervals for baseline elemental co-accumulation patterns among individual mice. Points represent median and lines represent 95% HPD.
