# Supplementary material for: Loss of Fmr1 reorganizes the multi-elemental composition across tissues in Fragile X Syndrome mice
Source: PLoS One. 2026 Jul 10;21(7):e0352693. doi: 10.1371/journal.pone.0352693 (PMC13354080; doi:10.1371/journal.pone.0352693)
Supplement: S5 File — Points represent median and lines represent 95% HPD. (DOCX) [file pone.0352693.s005.docx]

**Figure S5.** Genotype-specific forest plot displaying the posterior median residual correlations and corresponding 95% HPD intervals for all elemental pairs, isolated by genotype. Points represent median and lines represent 95% HPD.
