# Supplementary material for: Loss of Fmr1 reorganizes the multi-elemental composition across tissues in Fragile X Syndrome mice
Source: PLoS One. 2026 Jul 10;21(7):e0352693. doi: 10.1371/journal.pone.0352693 (PMC13354080; doi:10.1371/journal.pone.0352693)
Supplement: S7 File — Columns represent predicted median value, lower, and upper quartile, as well as observed median for each tissue, genotype, and element combination. (DOCX) [file pone.0352693.s007.docx]

**Table S2.** Verification matrix of model predictions. Columns represent predicted median value, lower, and upper quartile, as well as observed median for each tissue, genotype, and element combination.

| **Tissue** | **Element** | **WT_KO** | **Pred_Med** | **Pred_Lwr** | **Pred_Upr** | **Obs_Med** |
| --- | --- | --- | --- | --- | --- | --- |
| Cecal Contents | Ca | WT | -3.80 | -5.15 | -2.45 | -3.79 |
| Cecal Contents | Ca | KO | -3.81 | -5.45 | -2.35 | -3.76 |
| Cecal Contents | Cu | WT | -10.19 | -10.32 | -10.07 | -10.18 |
| Cecal Contents | Cu | KO | -10.15 | -10.29 | -10.01 | -10.19 |
| Cecal Contents | Fe | WT | -7.85 | -8.13 | -7.58 | -7.81 |
| Cecal Contents | Fe | KO | -7.72 | -8.01 | -7.41 | -7.72 |
| Cecal Contents | K | WT | -4.37 | -4.62 | -4.09 | -4.34 |
| Cecal Contents | K | KO | -4.27 | -4.57 | -3.96 | -4.31 |
| Cecal Contents | Mg | WT | -5.55 | -5.72 | -5.38 | -5.48 |
| Cecal Contents | Mg | KO | -5.49 | -5.68 | -5.27 | -5.47 |
| Cecal Contents | Mn | WT | -8.69 | -8.86 | -8.53 | -8.65 |
| Cecal Contents | Mn | KO | -8.67 | -8.85 | -8.48 | -8.69 |
| Cecal Contents | Na | WT | -4.37 | -4.64 | -4.06 | -4.36 |
| Cecal Contents | Na | KO | -4.30 | -4.64 | -3.99 | -4.34 |
| Cecal Contents | P | WT | -3.74 | -4.84 | -2.83 | -3.78 |
| Cecal Contents | P | KO | -3.81 | -4.94 | -2.65 | -3.75 |
| Cecal Contents | S | WT | -4.83 | -5.86 | -3.87 | -4.92 |
| Cecal Contents | S | KO | -4.96 | -6.10 | -3.84 | -4.87 |
| Cecal Contents | Zn | WT | -8.33 | -9.36 | -7.37 | -8.40 |
| Cecal Contents | Zn | KO | -8.31 | -9.40 | -7.16 | -8.26 |
| Feces | Ca | WT | -3.12 | -4.50 | -1.77 | -3.19 |
| Feces | Ca | KO | -3.26 | -4.80 | -1.75 | -3.31 |
| Feces | Cu | WT | -9.73 | -9.85 | -9.62 | -9.60 |
| Feces | Cu | KO | -9.88 | -10.02 | -9.75 | -9.91 |
| Feces | Fe | WT | -7.29 | -7.56 | -7.03 | -7.26 |
| Feces | Fe | KO | -7.40 | -7.68 | -7.08 | -7.37 |
| Feces | K | WT | -5.71 | -5.97 | -5.45 | -5.81 |
| Feces | K | KO | -5.43 | -5.75 | -5.14 | -5.23 |
| Feces | Mg | WT | -2.40 | -2.58 | -2.22 | -2.49 |
| Feces | Mg | KO | -2.70 | -2.92 | -2.52 | -2.71 |
| Feces | Mn | WT | -8.07 | -8.22 | -7.91 | -7.94 |
| Feces | Mn | KO | -8.14 | -8.33 | -7.97 | -8.26 |
| Feces | Na | WT | -4.58 | -4.88 | -4.31 | -4.67 |
| Feces | Na | KO | -5.05 | -5.38 | -4.71 | -4.94 |
| Feces | P | WT | -3.50 | -4.47 | -2.42 | -3.50 |
| Feces | P | KO | -3.60 | -4.74 | -2.46 | -3.57 |
| Feces | S | WT | -5.22 | -6.23 | -4.22 | -5.18 |
| Feces | S | KO | -5.20 | -6.29 | -4.04 | -5.18 |
| Feces | Zn | WT | -7.87 | -8.87 | -6.90 | -7.80 |
| Feces | Zn | KO | -8.13 | -9.22 | -6.98 | -8.16 |
| Fur | Ca | WT | -6.59 | -7.96 | -5.31 | -5.63 |
| Fur | Ca | KO | -5.77 | -7.37 | -4.29 | -5.01 |
| Fur | Cu | WT | -11.47 | -11.59 | -11.35 | -11.46 |
| Fur | Cu | KO | -11.48 | -11.62 | -11.35 | -11.37 |
| Fur | Fe | WT | -9.60 | -9.88 | -9.35 | -9.91 |
| Fur | Fe | KO | -9.65 | -9.94 | -9.34 | -10.07 |
| Fur | K | WT | -5.14 | -5.39 | -4.86 | -4.86 |
| Fur | K | KO | -5.62 | -5.92 | -5.29 | -5.56 |
| Fur | Mg | WT | -7.84 | -8.01 | -7.66 | -7.89 |
| Fur | Mg | KO | -7.94 | -8.15 | -7.75 | -7.96 |
| Fur | Mn | WT | -13.57 | -13.73 | -13.41 | -13.63 |
| Fur | Mn | KO | -13.61 | -13.79 | -13.43 | -13.71 |
| Fur | Na | WT | -6.63 | -6.92 | -6.35 | -6.90 |
| Fur | Na | KO | -7.00 | -7.35 | -6.68 | -7.27 |
| Fur | P | WT | -8.16 | -9.15 | -7.20 | -7.95 |
| Fur | P | KO | -8.18 | -9.31 | -7.01 | -8.08 |
| Fur | S | WT | -4.81 | -5.86 | -3.85 | -3.97 |
| Fur | S | KO | -4.47 | -5.56 | -3.28 | -3.62 |
| Fur | Zn | WT | -10.08 | -11.11 | -9.13 | -9.13 |
| Fur | Zn | KO | -9.59 | -10.70 | -8.43 | -8.78 |
| Olfactory Bulb | Ca | WT | -7.40 | -8.70 | -5.97 | -5.93 |
| Olfactory Bulb | Ca | KO | -6.48 | -8.01 | -4.90 | -4.81 |
| Olfactory Bulb | Cu | WT | -10.57 | -10.69 | -10.45 | -10.57 |
| Olfactory Bulb | Cu | KO | -10.60 | -10.73 | -10.46 | -10.58 |
| Olfactory Bulb | Fe | WT | -8.89 | -9.16 | -8.62 | -8.89 |
| Olfactory Bulb | Fe | KO | -8.74 | -9.05 | -8.44 | -8.76 |
| Olfactory Bulb | K | WT | -4.02 | -4.29 | -3.77 | -4.01 |
| Olfactory Bulb | K | KO | -4.11 | -4.39 | -3.79 | -4.05 |
| Olfactory Bulb | Mg | WT | -7.04 | -7.22 | -6.88 | -7.03 |
| Olfactory Bulb | Mg | KO | -6.87 | -7.07 | -6.68 | -6.96 |
| Olfactory Bulb | Mn | WT | -12.90 | -13.04 | -12.73 | -12.87 |
| Olfactory Bulb | Mn | KO | -12.82 | -13.00 | -12.64 | -12.88 |
| Olfactory Bulb | Na | WT | -5.14 | -5.43 | -4.87 | -5.13 |
| Olfactory Bulb | Na | KO | -5.17 | -5.52 | -4.86 | -5.16 |
| Olfactory Bulb | P | WT | -5.72 | -6.73 | -4.78 | -4.46 |
| Olfactory Bulb | P | KO | -5.62 | -6.82 | -4.54 | -4.10 |
| Olfactory Bulb | S | WT | -6.33 | -7.29 | -5.32 | -5.13 |
| Olfactory Bulb | S | KO | -6.31 | -7.43 | -5.19 | -4.93 |
| Olfactory Bulb | Zn | WT | -10.92 | -11.90 | -9.94 | -9.75 |
| Olfactory Bulb | Zn | KO | -10.77 | -11.94 | -9.71 | -9.45 |
| PMHTH | Ca | WT | -7.75 | -9.16 | -6.47 | -7.93 |
| PMHTH | Ca | KO | -9.10 | -10.62 | -7.57 | -7.57 |
| PMHTH | Cu | WT | -10.90 | -11.03 | -10.79 | -10.90 |
| PMHTH | Cu | KO | -10.91 | -11.06 | -10.78 | -10.94 |
| PMHTH | Fe | WT | -9.42 | -9.68 | -9.16 | -9.47 |
| PMHTH | Fe | KO | -9.32 | -9.65 | -9.03 | -9.32 |
| PMHTH | K | WT | -4.00 | -4.27 | -3.74 | -4.04 |
| PMHTH | K | KO | -4.01 | -4.35 | -3.72 | -3.98 |
| PMHTH | Mg | WT | -7.18 | -7.36 | -7.02 | -7.25 |
| PMHTH | Mg | KO | -7.28 | -7.48 | -7.08 | -7.28 |
| PMHTH | Mn | WT | -13.24 | -13.39 | -13.08 | -13.24 |
| PMHTH | Mn | KO | -13.22 | -13.40 | -13.04 | -13.18 |
| PMHTH | Na | WT | -5.13 | -5.44 | -4.86 | -5.12 |
| PMHTH | Na | KO | -5.13 | -5.47 | -4.80 | -5.11 |
| PMHTH | P | WT | -5.68 | -6.67 | -4.70 | -5.70 |
| PMHTH | P | KO | -6.68 | -7.85 | -5.59 | -5.38 |
| PMHTH | S | WT | -6.53 | -7.58 | -5.61 | -6.43 |
| PMHTH | S | KO | -7.11 | -8.33 | -6.09 | -6.16 |
| PMHTH | Zn | WT | -11.39 | -12.37 | -10.42 | -11.00 |
| PMHTH | Zn | KO | -11.71 | -12.87 | -10.62 | -10.78 |
| Striatum | Ca | WT | -6.43 | -7.71 | -5.10 | -6.52 |
| Striatum | Ca | KO | -6.41 | -7.94 | -4.79 | -6.38 |
| Striatum | Cu | WT | -10.40 | -10.52 | -10.27 | -10.35 |
| Striatum | Cu | KO | -10.39 | -10.53 | -10.26 | -10.41 |
| Striatum | Fe | WT | -9.29 | -9.54 | -9.02 | -9.26 |
| Striatum | Fe | KO | -9.18 | -9.51 | -8.89 | -9.21 |
| Striatum | K | WT | -4.27 | -4.53 | -4.01 | -4.26 |
| Striatum | K | KO | -4.22 | -4.52 | -3.92 | -4.22 |
| Striatum | Mg | WT | -5.85 | -6.03 | -5.67 | -5.88 |
| Striatum | Mg | KO | -5.79 | -6.01 | -5.60 | -5.64 |
| Striatum | Mn | WT | -12.97 | -13.12 | -12.80 | -12.90 |
| Striatum | Mn | KO | -13.01 | -13.19 | -12.83 | -13.02 |
| Striatum | Na | WT | -4.28 | -4.56 | -4.00 | -4.24 |
| Striatum | Na | KO | -4.20 | -4.52 | -3.86 | -4.18 |
| Striatum | P | WT | -3.98 | -4.88 | -2.98 | -3.99 |
| Striatum | P | KO | -3.93 | -5.11 | -2.79 | -3.92 |
| Striatum | S | WT | -4.78 | -5.76 | -3.85 | -4.81 |
| Striatum | S | KO | -4.73 | -5.89 | -3.63 | -4.73 |
| Striatum | Zn | WT | -9.52 | -10.52 | -8.62 | -9.56 |
| Striatum | Zn | KO | -9.60 | -10.71 | -8.48 | -9.66 |
