# Supplementary material for: Loss of Fmr1 reorganizes the multi-elemental composition across tissues in Fragile X Syndrome mice
Source: PLoS One. 2026 Jul 10;21(7):e0352693. doi: 10.1371/journal.pone.0352693 (PMC13354080; doi:10.1371/journal.pone.0352693)
Supplement: S8 File — Values represent the Interquartile Range (IQR) and Standard Deviation (SD) of the model-derived residual error on the Additive Log-Ratio (ALR) scale, isolated by genotype. (DOCX) [file pone.0352693.s008.docx]

**Table S3.** Post-hoc assessment of multivariate residual dispersion and homoscedasticity. Values represent the Interquartile Range (IQR) and Standard Deviation (SD) of the model-derived residual error on the Additive Log-Ratio (ALR) scale, isolated by genotype.

| **WT_KO** | **Residual_IQR** | **Residual_SD** |
| --- | --- | --- |
| KO | 0.17 | 0.19 |
| WT | 0.21 | 0.23 |
