# Supplementary material for: Loss of Fmr1 reorganizes the multi-elemental composition across tissues in Fragile X Syndrome mice
Source: PLoS One. 2026 Jul 10;21(7):e0352693. doi: 10.1371/journal.pone.0352693 (PMC13354080; doi:10.1371/journal.pone.0352693)
Supplement: S9 File — Values represent the Interquartile Range (IQR) and Standard Deviation (SD) of the model-derived residual error on the Additive Log-Ratio (ALR) scale, isolated by tissue type. (DOCX) [file pone.0352693.s009.docx]

**Table S4.** Post-hoc assessment of multivariate residual dispersion and homoscedasticity. Values represent the Interquartile Range (IQR) and Standard Deviation (SD) of the model-derived residual error on the Additive Log-Ratio (ALR) scale, isolated by tissue type.

| **Tissue** | **Residual_IQR** | **Residual_SD** |
| --- | --- | --- |
| Cecal Contents | 0.155 | 0.157 |
| Feces | 0.197 | 0.240 |
| Fur | 0.154 | 0.145 |
| Olfactory Bulb | 0.119 | 0.113 |
| PMHTH | 0.115 | 0.146 |
| Striatum | 0.528 | 0.392 |
