# Supplementary material for: Loss of Fmr1 reorganizes the multi-elemental composition across tissues in Fragile X Syndrome mice
Source: PLoS One. 2026 Jul 10;21(7):e0352693. doi: 10.1371/journal.pone.0352693 (PMC13354080; doi:10.1371/journal.pone.0352693)
Supplement: Attachment — Submitted filename: Ionome PLoS one_reviewer.docx [file pone.0352693.s011.docx]

We thank the reviewers and editor for their suggestions for revision for our manuscript. We have made substantial changes including a new analysis pipeline that has significantly strengthened the manuscript. We have addressed reviewer’s concerns point by point in the document below.

Reviewer #1: This is a very interesting study measuring the multivariate balance of 10 elements in tissues of WT and Fmr1KO mice. The authors compared ion composition of gut, brain, fur and feces in WT and Fmr1KO mice. Differences are found as a function of brain region and genotype. Of note, higher iron is found in the olfactory bulb of Fmr1KO and higher magnesium and zinc in the feces of WT mice. The authors discuss the findings considering autism spectrum disorders. Overall, the study of ionomics is important, and relatively new, to the fragile X field potentially offering new biomarkers.

Thank you for the kind review and valuable feedback. We have incorporated suggestions and addressed concerns point by point below.

Issues to Address:

Abstract
Line 13: recommend changing “and ion flux” to “including mRNAs that code for proteins regulating ion flux”. - Updated

Lines 13-14: The statement, “a comprehensive comparison of elemental balance between FXS genotypes and tissues remains absent from the literature”, does not seem fair considering the work by Talvio et al measuring Na, Mg, P, K, Mn, Fe and Cu in cortex, cerebellum, liver, spleen and heart.

Updated to read

“However, there are few studies measuring the elemental balance between FXS genotypes and tissues.”

Introduction
Line 72: Since there is no global mechanism enforcing a fixed total elemental constant, why would elements be constrained to a constant sum? Aren’t elements regulated independently, i.e., sodium via kidneys and aldosterone; calcium via bone remodeling, PTH and vitamin D; and iron via absorption and recycling? In a related publication by Talvio and colleagues, they compared absolute amounts of elements in WT versus Fmr1KO mice.

We agree that biological systems do not possess a single physiological mechanism enforcing a fixed total elemental inventory across tissues. Individual elements are regulated through distinct physiological pathways operating at different rates across cells and tissues (e.g., renal handling of Na, hormonal regulation of Ca and P, iron absorption and recycling).

Our use of a compositional framework does not assume coordinated physiological regulation toward a biologically fixed elemental total. Rather, compositional analysis reflects the structure of multielement measurements themselves. Because tissues are open dynamical systems with simultaneous elemental uptake, storage, transport, utilization, and excretion occurring at different rates for different elements, interpretation of any single elemental abundance is necessarily contextualized by the relative abundances of other measured elements within the same sample. Compositional approaches are designed to account for these interdependencies and avoid artifacts associated with treating multielement measurements as unconstrained independent variables (Aitchison 1986; Greenacre et al. 2021). We revised the manuscript to clarify that our use of compositional data analysis arises from the relative nature of multielement measurements rather than from an assumption of a biologically fixed elemental total.

Methods
More detail should be added on animal husbandry. Were littermate mice used? What was the diet/bedding/etc. that may have affected mineral levels.

More information has been included on the husbandry and diet/bedding. See below:

“All mice were on a 12-hour light cycle (6 AM- 6 PM) and received the same chow (LabDiet 500, Lab Supply, Fort Worth, TX) 1 and bedding (alpha-dri, Innovive, San Diego, CA) and additional nestlets (cotton NST, Lab Supply, Fort Worth, TX).” We have also included information on husbandry including that all animals were littermates and therefore data reflect animals from two cages per genotype.

Discussion
Can the authors add to the discussion: (1) any limitations associated with the use of isoflurane that may affect mineral, particularly iron, levels?

We have added the following statement in our discussion section:

“Although isoflurane exposure was applied uniformly across experimental groups, previous studies have reported that inhalation of anesthetics can affect iron homeostasis, ferroptosis-related pathways, intracellular metabolite diffusion, and mineral-regulatory processes (Wang et al. 2019; Miao et al. 2024). Together, these findings suggest that isoflurane may affect elemental and metabolic homeostasis and therefore represents a potential confounding factor when interpreting tissue mineral composition data, particularly iron-related measurements, and is an important difference between our work and previous published literature (Talvio et al. 2021).”

(2) compare/contrast methods with Talvio such as CO2 versus isoflurane (see above). (3) Discuss the large difference in absolute mineral content between the current study and the Talvio study Table 3. Talvio has data for cortex and cerebellum, and the authors have PMHTH and olfactory brain. It looks like about a 10-fold difference for iron, and other elements also exhibit large differences. (4) Discuss future directions including the need to assess labile mineral content. The extent of iron toxicity depends on localization of the iron within the cell and its biochemical form.

We have added the following to the discussion:

(3) While previous work has also measured elemental differences in FXS, direct quantitative comparison between the present study and others should be interpreted cautiously because the studies differed substantially in tissue selection, analytical methodology, and normalization strategy. Talvio et al. quantified absolute elemental concentrations (µg/g wet weight) in cortex, cerebellum, heart, liver, and spleen using ICP-MS, whereas our study analyzed dried tissue samples using ICP-OES, which is less sensitive, within a compositional framework focused on relative multielement organization across olfactory bulb, striatum, PMHTH, fur, feces, and cecal contents. Additionally, the studies differed in tissue preparation and experimental workflow, including anesthetic exposure, tissue pooling strategy, drying procedures, digestion workflows, and elemental normalization approaches. These methodological differences are expected to influence absolute elemental concentrations, particularly for redox-sensitive elements such as iron. Importantly, Talvio et al. similarly emphasized cautious interpretation due to limited statistical power and multiple comparisons (Talvio et al. 2021). Our study was designed to evaluate whether loss of Fmr1 alters relative multielement organization across neural and somatic tissues. In this compositional framework, interpretation primarily depends on relative elemental relationships rather than direct equivalence of absolute concentrations.

(4) “Even though, in this study we measured overall relative concentration of an element in different neural as well as somatic tissues, future studies should assess not only total elemental abundance but also labile mineral content. The reason behind this logic is total tissue mineral levels do not unveil whether an element is biologically in active form, safely stored, protein-bound, redox-active, or localized within specific cellular compartments in tissues. This especially carries crucial insights for iron, because iron toxicity depends strongly on its biochemical form and intracellular localization. For example- ferritin-bound or transferrin-bound iron is relatively protected, whereas labile Fe²⁺ can promote Fenton chemistry, oxidative stress, lipid peroxidation, and ferroptosis (Kakhlon and Cabantchik, 2002). Thus, two tissues may show similar total iron concentrations, yet differ substantially in toxicity or physiological impact if one contains a larger labile/redox-active iron pool. Therefore, measuring only total tissue iron could not fully capture biologically relevant alterations in iron homeostasis in our study.

For the other elements measured in this study — their labile pools may provide more mechanistic insight than total elemental concentration alone. For example, labile Ca²⁺ regulates ion transport, secretion, epithelial signaling, and cellular stress responses (Berridge et al. 2003); labile Zn²⁺ functions as a signaling ion (De Leon-Rodriguez et al. 2012); and labile Cu and Mn may influence redox biology, mitochondrial function, and oxidative stress (Lindahl and Moore 2016).

Future studies should investigate labile metal pools within the gut lumen and microbial environment, because total elemental abundance in luminal contents may not accurately reflect the fraction of minerals that is biologically available to microbes or host epithelial cells. Since the concept of “Labile metals” refers to exchangeable, bioavailable metal ions that actively participate in microbial metabolism, redox reactions, signaling, and host–microbe interactions rather than metals that are tightly bound or inertly stored. Previous studies also emphasize that bacteria dynamically regulate labile metal pools to maintain metabolic homeostasis, oxidative stress resistance, and nutrient acquisition (Helmann 2025).

This is particularly relevant to our study because we quantified elemental concentrations in intestinal luminal contents, where metals are simultaneously influenced by diet, microbial utilization, epithelial transport, mucus interactions, and host inflammatory responses (Hood and Skaar 2012; Lopez and Skaar 2018). Therefore, future studies should examine not only total elemental levels but also the biologically active and compartment-specific metal pools, as these may better explain how altered mineral homeostasis contributes to gut microbiome changes and other neural as well as peripheral phenotypes in FXS.

Therefore, future work is needed to combine bulk elemental profiling with approaches that measure bioavailable and compartment-specific mineral pools. This would help determine whether altered mineral composition in FXS reflects changes in total abundance, altered intracellular distribution, or shifts in the biologically reactive mineral pools most likely to affect mitochondrial metabolism, oxidative stress and cellular toxicity.

(5) The olfactory bulb is one of the most iron sensitive regions of the brain. The authors could discuss how their results fit with the Fmr1KO mouse literature on olfactory bulb outcomes.

We subsequently shifted analytical strategies due to suggestions to include mixed models, and updated results do not reflect previously observed differences.

Reviewer #2: This study by Alam et al. carries out a comparison of elemental ion balance between male Fmr1 KO mice and WT mice of 3-5 months of age, sampling from several brain regions and somatic tissue. Given prior findings of ionic dysregulation in FXS literature, these results and approach to evaluate global ionomics is of interest to a broader FXS and ASD audience. The measurements across the brain regions and gut (cecal and fecal) is novel, the analytical framework useful and the potential for multi domain comparisons intriguing.
Given the novelty of assessing applicability of 'ionomic' analysis to ASD and FXS literature, I would support publication, if the following concerns are addressed.
Broadly, the major concerns are analytical discrepancies (which seem typographical errors) and unshared data which raise concerns. Moderate concerns are incorrect citations, toning down the conclusion of the writing (especially) in the discussion which should be more precise and rigorous.

Thank you for your insights and suggestions which we have incorporated below in a point-by-point response. We have since made sure that all of our data is now available – see updated statement with links to the data.

Major Concern 1)
1. Although, the data availability statement states that data is available and relevant data within the manuscript, I could not find – see updated statement with links to data
1) average element concentrations for each sample.
2) the ALR-transformed values.
3) details about both the missing values (which samples, tissue and genotype), and tables of imputed numbers. This is especially critical for Zn as there were 7 missing values and Zn was one of the elements that was significantly dysregulated in a genotype specific manner.

We corrected the number of missing Zn values to 8 and specified the sample information. Additionally, we have provided the raw data csv along with code to produce all analyses with these revisions, including initial imputation and alr-transformation.

The only available data set was the Mean SEM in Table S8 and S9 and here there a couple of glaring inconsistencies,
1. Table S9- large numbers. For example, striatum WT is 74.6 ± 0.006 µg/mg whereas KO is 0.067 ± 0.004 µg/mg. (Is this a typo? 0.074 would match their results). Similarly, fur calcium is transcribed as WT 39.2, KO 6.2. These numbers are unusually large.
2. Table S8, if derived from S9 (by normalizing to Fv), do not reflect these proportions.
The results in the table S8, for most parts match the conclusions. However, none of the mean difference findings reach significance when tested from Table S8 values (since the test were on ALR transformed values). Without the availability of ALR transformed values, which is missing, these results cannot be verified.

Erroneously large values were typos in previous tables - we thank the reviewer for their diligent and careful eye.

Methodological concerns.
Study design:
1. Please expand on the breeding strategy (Hom x Hom, Hom x WT) and housing information (were mice single or grouped housed). Gut microbiome would normalize if grouped housed and cage effects would need to be considered.

Our new comprehensive Bayesian multivariate mixed model approach should account for this variance as well. In our analysis the contribution of individual animal variation was low, and we expect this would be similar for group-housing effects. We have included more information in the methods on housing (group, etc. and breeding scheme). All our data were from group-housed individuals which results in only two cages per genotype.

2. Please state in the methods that all samples are from the same individual (if this is the case)
If samples are from within an individual mouse, it is unclear why the authors did not model this into their analysis. The MANOVA pools all observations together, but those observations are not truly independent, violating the independence assumption. A multivariate mixed model would have been more appropriate. If the authors did try this, and did not have sufficient power, this should be acknowledged in the results.

In response to this and the subsequent comment, we have wholly shifted our analytical framework to a Bayesian multivariate mixed model to allow better inference of variance partitioning.

3. The authors missed an opportunity to compare within individual, cross-tissue correlations (which would have been possible with a mixed model). This would have been a good comparison to have given the individual variabilities seen in Fmr1 KO mice.
4. The MANOVA's are multiple comparisons, and multi-measure corrections are absent.

MANOVA’s and PCA have subsequently been replaced with a Bayesian mixed-modeling framework which also addresses within individual correlations.

5. Although, the authors do justify the use of male mice in the methods, sex differences (especially in behavior) have been observed, and would should be acknowledged as a caveat.

We added this to the discussion:

“Additionally we only used male mice in our study, previous work has shown that there are sex differences physiologically (Chawla and McCullagh 2022; Smith et al. 2021) and behaviorally (Petroni et al. 2022) in FXS mice suggesting that sex may also be an important factor for future work.

Tissue collection: The authors mention the collection of several regions, but it's unclear why these regions were excluded from the results. Similarly, the rationale for mixing anatomically diverse regions, with variable cellular composition was not explained.

We have included additional information to clarify collection of tissues.

1. I am surprised the authors did not evaluate serum levels. That would have been informative and complemented their other measurements. Especially, since the authors seem to be directly extracting tissue (with no ACSF or saline perfusions which would normalize ionic balances across mice), tissue specific dysregulation could be a reflection of circulatory changes.

We agree that serum elemental measurements would have provided valuable complementary information for distinguishing systemic versus tissue-associated elemental alterations. In the present study, tissues were collected without saline or ACSF perfusion, and therefore measured elemental composition may partially reflect residual vascular or circulatory contributions in addition to tissue-associated ionomic organization. We have now clarified this limitation in the discussion section and revised our interpretation accordingly.

2. For clarity, consider re-ordering the order of sample collection. I am assuming the fecal collection was done first, followed by brain, cecal and fur.

We have reordered the tissue collection section and revised it.

3. I may have missed it, but there is no records of ages of mice from which samples were collected. It is unclear if ionic compositions are consistent across age and if this was assessed.

We have included information regarding age in the methods and added age as a limiting factor in the discussion. Mice used in this study were between 99–156 days of age at the time of sample collection. While all experimental groups were age-matched as closely as possible, we acknowledge that age-related physiological and metabolic changes could potentially influence tissue elemental composition (Morel et al 2022). Assessing age-associated variability in ionic composition across tissues represents an important future direction for larger cohort studies specifically to evaluate age-dependent effects.

Figures
Figure 1- It would be clearer if the genotype were identified by symbols and tissue by color. That would give a good sense of the genotype differences.
Figure 2- Maybe the authors could change the symbol of the entries, or state that the black dots indicate outliers. Otherwise it gets confusing.

The first two figures have both been changed in line with new analysis.

Confusing statements or references:
1. Line 13- 'comprehensive' is an overclaim. The study is a good 'proof of principle' of assessing ionomics as a viable and informative approach in FXS. A comprehensive study would require a lot more statistical power and precision in tissue extraction.

We have updated accordingly.
2. Line 28- typo. Salcedo-Arellano et al., is 2020 not 2023. – fixed
3. Line 40 This argument is confusing and logic inverted. – fixed
1. The references they use are bulk RNAseq and proteomic studies. These do not track single molecular pathways but capture system wide cellular perturbations. The ionome would reflect altered cellular processing (especially brain ionome), and hence would be a good complement but not be a substitute to transcriptomic or proteomic approaches.

The sentence has been changed to reflect comments and now reads: “Responses of the ionome to genetic perturbations have been used to advance understanding of various phenotypes (Huang and Salt, 2016) including disease (Cabral et al., 2021; Dubey et al., 2020; Sarafanov et al., 2011; Zhang et al., 2020; Zhang et al., 2023) which could be considered complementary to other techniques such as transcriptomics (Ding et al., 2020; Ebrahimiazar et al., 2025, Jung et al., 2023) and proteomic analyses (Abbasi et al., 2024; Gao et al., 2023 (Tang et al. 2015)).

2. Donnard 2022 is a single cell RNAseq study, not a bulk RNAseq study. - removed
3. Missing references. For the bulk RNAseq Jung et al., 2023 PMID: 38048343 would support their results. – added
4. For proteomics Tang et al., PMID 26307763 should also be cited. – added
4. Line 45 Fmr1 should be italicized. - fixed
5. Line 49: Stefani 2004 does not link FMRP to identity of transcripts. HIT-clips studies Darnell 2011 and Maurin 2018 would be more appropriate references. Similarly Zhou et al., 2025 is not a neuronal paper, but a paper about the role of FMRP in tumor microenvironment. – updated
6. Line 79; location of the reference (Greenacre 2021) is confusing as it seems to imply the reference refers to elemental reorganization in FXS. Maybe the placement was meant to be before? -removed
7. Line 228: '... indicated ionomic similarity between genotypes, although PMHTH and striatum did not overlap', are the authors talking about genotype or tissue type comparisons?

We have updated the text to read:

In this study, multivariate analyses demonstrated that elemental composition patterns were driven mainly by tissue type rather than genotype, with samples clustering primarily according to their tissue origin. However, PMHTH and striatum showed some separation between WT and KO animals, along with differences in variability, suggesting that Fmr1 deficiency may alter elemental organization in specific brain regions. The distinct positioning of PMHTH compared with other tissues may also reflect the complex and heterogeneous nature of this combined brain-region sample, in addition to normal tissue-specific differences in elemental composition.

8. Line 237 (Kooy, 2003) The authors are conflating human animal model face-validity with variability in rodent models. A better reference for the latter is Kat 2022 (PMID: 35690123) – we have since changed this section to reflect updated results.

9. Line 239, variance in 'both' not 'either' - we have since changed this section to reflect updated results.

10. Line 245: "FXS pathologies may also be expected ..." ionic dysregulation and neural excitability have been observed repeatedly in FXS, across several papers as highlighted in the previous line. Secondly, the references Napoli 2016 is about Zn, which is not affected in the brain. Similarly, D’Antoni et al., 2024, does not link FXS to trace elements or metalloenzymes. There are several links of FXS to metalloproteases and ECM (especially MMP9 among others). Please modify.

We have modified this section to reflect updated findings.

11. Line 268, The sentence is confusing and is unclear what the authors are trying to link. They refer to zinc-binding motifs (maybe proteins with zinc-binding motifs) and FMRP-pathways? Secondly, Edbauer 2010 is not an appropriate reference since as it does not link Zn and FXS).

We have updated this section to reflect updated analyses

12. Line 289, I disagree with the authors assertion of ionomics as a reverse genetics lens, to provide mechanistic insights, especially in complex multi-cellular tissue such as the brain. The papers the authors cite are papers from homogenous tissue or cell lines, where perturbing genes and linking to elements is possible. However, recent studies with cell specific manipulations (Fmrp knockouts, FMRP reexpression, or RNA translatome studies) suggest cell specific roles of FMRP (across neurons, interneurons and glia). Unless the authors are suggesting cell specific ionome (with cell-sorting), global ionome signals will be hard to resolve to the cellular source (genomic 'haystacks' are now cellular 'haystacks'). At best, the current iteration of ionome would be as a phenotypic tool and its applicability to tissue specific ionomic profiling in identifying tissue-element disruptions I agree. It might also be possible to link behavioral deficits to fecal elemental composition, would potentially enable biomarker discovery (which the authors state).

To provide clarity and not overstate results, we have removed this sentence.

13. Line 302, similar to my above comment, integrating global ionome with global transcriptome, proteome screens to identify cellular mechanisms, especially with cell specific dysregulation, is challenging.

Have updated to reflect challenge and rewritten section to not overstate the value of ionomics.
