# Supplementary material for: Loss of Fmr1 reorganizes the multi-elemental composition across tissues in Fragile X Syndrome mice
Source: PLoS One. 2026 Jul 10;21(7):e0352693. doi: 10.1371/journal.pone.0352693 (PMC13354080; doi:10.1371/journal.pone.0352693)
Supplement: Peer review history

# Peer review: Decision Letter 0

**DOI:** 10.1371/journal.pone.0352693.r001

**Authors:** Nien-Pei Tsai

16 Apr 2026

PONE-D-26-15087

Loss of Fmr1 reorganizes the multi-elemental composition of neural and somatic tissues in Fragile X Syndrome mice

PLOS One

Dear Dr. McCullagh,

Thank you for submitting your manuscript to PLOS ONE. After careful consideration, we feel that it has merit but does not fully meet PLOS ONE’s publication criteria as it currently stands. Therefore, we invite you to submit a revised version of the manuscript that addresses the points raised during the review process.

Please submit your revised manuscript by May 31 2026 11:59PM. If you will need more time than this to complete your revisions, please reply to this message or contact the journal office at plosone@plos.org. When you're ready to submit your revision, log on to https://www.editorialmanager.com/pone/ and select the 'Submissions Needing Revision' folder to locate your manuscript file.

Please include the following items when submitting your revised manuscript:

- A letter that responds to each point raised by the academic editor and reviewer(s). You should upload this letter as a separate file labeled 'Response to Reviewers'.
- A marked-up copy of your manuscript that highlights changes made to the original version. You should upload this as a separate file labeled 'Revised Manuscript with Track Changes'.
- An unmarked version of your revised paper without tracked changes. You should upload this as a separate file labeled 'Manuscript'.

If you would like to make changes to your financial disclosure, please include your updated statement in your cover letter. Guidelines for resubmitting your figure files are available below the reviewer comments at the end of this letter.

If applicable, we recommend that you deposit your laboratory protocols in protocols.io to enhance the reproducibility of your results. Protocols.io assigns your protocol its own identifier (DOI) so that it can be cited independently in the future. For instructions see: https://journals.plos.org/plosone/s/submission-guidelines#loc-laboratory-protocols. Additionally, PLOS ONE offers an option for publishing peer-reviewed Lab Protocol articles, which describe protocols hosted on protocols.io. Read more information on sharing protocols at https://plos.org/protocols?utm_medium=editorial-email&utm_source=authorletters&utm_campaign=protocols.

As the corresponding author, your ORCID iD is verified in the submission system and will appear in the published article. PLOS supports the use of ORCID, and we encourage all coauthors to register for an ORCID iD and use it as well. Please encourage your coauthors to verify their ORCID iD within the submission system before final acceptance, as unverified ORCID iDs will not appear in the published article. *Only*  the individual author can complete the verification step; PLOS staff *cannot*  verify ORCID iDs on behalf of authors.

We look forward to receiving your revised manuscript.

Kind regards,

Nien-Pei Tsai, PhD

Academic Editor

PLOS One

Journal Requirements:

When submitting your revision, we need you to address these additional requirements.

1.Please ensure that your manuscript meets PLOS ONE's style requirements, including those for file naming. The PLOS ONE style templates can be found at

https://journals.plos.org/plosone/s/file?id=wjVg/PLOSOne_formatting_sample_main_body.pdf and

https://journals.plos.org/plosone/s/file?id=ba62/PLOSOne_formatting_sample_title_authors_affiliations.pdf

2. Please note that PLOS One has specific guidelines on code sharing for submissions in which author-generated code underpins the findings in the manuscript. In these cases, we expect all author-generated code to be made available without restrictions upon publication of the work. Please review our guidelines at https://journals.plos.org/plosone/s/materials-and-software-sharing#loc-sharing-code and ensure that your code is shared in a way that follows best practice and facilitates reproducibility and reuse.

3. Please note that funding information should not appear in any section or other areas of your manuscript. We will only publish funding information present in the Funding Statement section of the online submission form. Please remove any funding-related text from the manuscript.

4. Thank you for stating in your Funding Statement:

“This work was partially funded by NIH COBRE pilot award through the Oklahoma Center for Microbiome Research to EAM.”

Please provide an amended statement that declares *all* the funding or sources of support (whether external or internal to your organization) received during this study, as detailed online in our guide for authors at http://journals.plos.org/plosone/s/submit-now.  Please also include the statement “There was no additional external funding received for this study.” in your updated Funding Statement.

Please include your amended Funding Statement within your cover letter. We will change the online submission form on your behalf.

5. Please expand the acronym “NIH COBRE” (as indicated in your financial disclosure) so that it states the name of your funders in full.

This information should be included in your cover letter; we will change the online submission form on your behalf.

6. Thank you for stating the following financial disclosure:

“This work was partially funded by NIH COBRE pilot award through the Oklahoma Center for Microbiome Research to EAM.”

Please state what role the funders took in the study.  If the funders had no role, please state: "The funders had no role in study design, data collection and analysis, decision to publish, or preparation of the manuscript."

If this statement is not correct you must amend it as needed.

Please include this amended Role of Funder statement in your cover letter; we will change the online submission form on your behalf.

7. We note that your Data Availability Statement is currently as follows: All relevant data are within the manuscript and in Supporting Information files.

Please confirm at this time whether or not your submission contains all raw data required to replicate the results of your study. Authors must share the “minimal data set” for their submission. PLOS defines the minimal data set to consist of the data required to replicate all study findings reported in the article, as well as related metadata and methods (https://journals.plos.org/plosone/s/data-availability#loc-minimal-data-set-definition).

For example, authors should submit the following data:

- The values behind the means, standard deviations and other measures reported;

- The values used to build graphs;

- The points extracted from images for analysis.

Authors do not need to submit their entire data set if only a portion of the data was used in the reported study.

If your submission does not contain these data, please either upload them as Supporting Information files or deposit them to a stable, public repository and provide us with the relevant URLs, DOIs, or accession numbers. For a list of recommended repositories, please see https://journals.plos.org/plosone/s/recommended-repositories.

If there are ethical or legal restrictions on sharing a de-identified data set, please explain them in detail (e.g., data contain potentially sensitive information, data are owned by a third-party organization, etc.) and who has imposed them (e.g., an ethics committee). Please also provide contact information for a data access committee, ethics committee, or other institutional body to which data requests may be sent. If data are owned by a third party, please indicate how others may request data access.

8. When completing the data availability statement of the submission form, you indicated that you will make your data available on acceptance. We strongly recommend all authors decide on a data sharing plan before acceptance, as the process can be lengthy and hold up publication timelines. Please note that, though access restrictions are acceptable now, your entire data will need to be made freely accessible if your manuscript is accepted for publication. This policy applies to all data except where public deposition would breach compliance with the protocol approved by your research ethics board. If you are unable to adhere to our open data policy, please kindly revise your statement to explain your reasoning and we will seek the editor's input on an exemption. Please be assured that, once you have provided your new statement, the assessment of your exemption will not hold up the peer review process.

9. Please include captions for your Supporting Information files at the end of your manuscript, and update any in-text citations to match accordingly. Please see our Supporting Information guidelines for more information: http://journals.plos.org/plosone/s/supporting-information.

10. If the reviewer comments include a recommendation to cite specific previously published works, please review and evaluate these publications to determine whether they are relevant and should be cited. There is no requirement to cite these works unless the editor has indicated otherwise.

Additional Editor Comments:

While both reviewers appreciate the importance of this study to FXS field, Reviewer 2 has raised concerns about the clarity of data presentation and statistical analysis performed in this study. The authors are recommended to consider the suggestions provided by both reviewers when revising their manuscript.

[Note: HTML markup is below. Please do not edit.]

Reviewers' comments:

Reviewer's Responses to Questions

**Comments to the Author**

1. Is the manuscript technically sound, and do the data support the conclusions?

The manuscript must describe a technically sound piece of scientific research with data that supports the conclusions. Experiments must have been conducted rigorously, with appropriate controls, replication, and sample sizes. The conclusions must be drawn appropriately based on the data presented.

Reviewer #1: Yes

Reviewer #2: Partly

**********

2. Has the statistical analysis been performed appropriately and rigorously?

Reviewer #1: I Don't Know

Reviewer #2: No

**********

3. Have the authors made all data underlying the findings in their manuscript fully available?

The PLOS Data policy requires authors to make all data underlying the findings described in their manuscript fully available without restriction, with rare exception (please refer to the Data Availability Statement in the manuscript PDF file). The data should be provided as part of the manuscript or its supporting information, or deposited to a public repository. For example, in addition to summary statistics, the data points behind means, medians and variance measures should be available. If there are restrictions on publicly sharing data—e.g. participant privacy or use of data from a third party—those must be specified.

Reviewer #1: Yes

Reviewer #2: No

**********

4. Is the manuscript presented in an intelligible fashion and written in standard English?

PLOS ONE does not copyedit accepted manuscripts, so the language in submitted articles must be clear, correct, and unambiguous. Any typographical or grammatical errors should be corrected at revision, so please note any specific errors here.

Reviewer #1: Yes

Reviewer #2: No

**********

5. Review Comments to the Author

Please use the space provided to explain your answers to the questions above. You may also include additional comments for the author, including concerns about dual publication, research ethics, or publication ethics. (Please upload your review as an attachment if it exceeds 20,000 characters)

Reviewer #1: This is a very interesting study measuring the multivariate balance of 10 elements in tissues of WT and Fmr1KO mice. The authors compared ion composition of gut, brain, fur and feces in WT and Fmr1KO mice. Differences are found as a function of brain region and genotype. Of note, higher iron is found in the olfactory bulb of Fmr1KO and higher magnesium and zinc in the feces of WT mice. The authors discuss the findings considering autism spectrum disorders. Overall, the study of ionomics is important, and relatively new, to the fragile X field potentially offering new biomarkers.

Issues to Address:

Abstract

Line 13: recommend changing “and ion flux” to “including mRNAs that code for proteins regulating ion flux”.

Lines 13-14: The statement, “a comprehensive comparison of elemental balance between FXS genotypes and tissues remains absent from the literature”, does not seem fair considering the work by Talvio et al measuring Na, Mg, P, K, Mn, Fe and Cu in cortex, cerebellum, liver, spleen and heart.

Introduction

Line 72: Since there is no global mechanism enforcing a fixed total elemental constant, why would elements be constrained to a constant sum? Aren’t elements regulated independently, i.e., sodium via kidneys and aldosterone; calcium via bone remodeling, PTH and vitamin D; and iron via absorption and recycling? In a related publication by Talvio and colleagues, they compared absolute amounts of elements in WT versus Fmr1KO mice.

Methods

More detail should be added on animal husbandry. Were littermate mice used? What was the diet/bedding/etc. that may have affected mineral levels.

Discussion

Can the authors add to the discussion: (1) any limitations associated with the use of isoflurane that may affect mineral, particularly iron, levels? (2) compare/contrast methods with Talvio such as CO2 versus isoflurane. (3) Discuss the large difference in absolute mineral content between the current study and the Talvio study Table 3. Talvio has data for cortex and cerebellum, and the authors have PMHTH and olfactory brain. It looks like about a 10-fold difference for iron, and other elements also exhibit large differences. (4) Discuss future directions including the need to assess labile mineral content. The extent of iron toxicity depends on localization of the iron within the cell and its biochemical form. (5) The olfactory bulb is one of the most iron sensitive regions of the brain. The authors could discuss how their results fit with the Fmr1KO mouse literature on olfactory bulb outcomes.

Reviewer #2: This study by Alam et al. carries out a comparison of elemental ion balance between male Fmr1 KO mice and WT mice of 3-5 months of age, sampling from several brain regions and somatic tissue. Given prior findings of ionic dyregulation in FXS literature, these results and approach to evaluate global ionomics is of interest to a broader FXS and ASD audience. The measurements across the brain regions and gut (ceacal and fecal) is novel, the analytical framework useful and the potential for multi domain comparisons intriguing.

Given the novelty of assessing applicability of 'ionomic' analysis to ASD and FXS literature, I would support publication, if the following concerns are addressed.

Broadly, the major concern are analytical discrepancies (which seem typographical errors) and unshared data which raise concerns. Moderate concerns are incorrect citations, toning downs conclusion the writing (especially) in the discussion which should be more precise rigorous.

Major Concern 1)

1. Although, the data availability statement states that data is available and relevant data within the manuscript, I could not find

1) average element concentrations for each sample.

2) the ALR-transformed values.

3) details about both the missing values (which samples, tissue and genotype), and tables of imputed numbers. This is especially critical for Zn as there were 7 missing values and Zn was one of the elements that was significantly dysregulated in a genotype specific manner.

The only available data set was the Mean SEM in Table S8 and S9 and here there a couple of glaring inconsistencies,

1. Table S9- large numbers. For example, striatum WT is 74.6 ± 0.006 µg/mg whereas KO is 0.067 ± 0.004 µg/mg. (Is this a typo? 0.074 would match their results). Similarly, fur calcium is transcribed as WT 39.2, KO 6.2. These numbers are unusually large.

2. Table S8, if derived from S9 (by normalizing to Fv), do not reflect these proportions.

The results in the table S8, for most parts match the conclusions. However, none of the mean difference findings reach significance when tested from Table S8 values (since the test were on ALR transformed values). Without the availability of ALR transformed values, which is missing, these results cannot be verified.

Methodological concerns.

Study design:

1. Please expand on the breeding strategy (Hom x Hom, Hom x WT) and housing information (were mice single or grouped housed). Gut microbiome would normalize if grouped housed and cage effects would need to be considered.

2. Please state in the methods that all samples are from the same individual (if this is the case)

If samples are from within an individual mouse, it is unclear why the authors did not model

this into their analysis. The MANOVA pools all observations together, but those observations are not truly independent, violating the independence assumption. A multivariate mixed model would have been more appropriate. If the authors did try this, and did not have sufficient power, this should be acknowledged in the results.

3. The authors missed an opportunity to compare within individual, cross-tissue correlations (which would have been possible with a mixed model). This would have been a good comparison to have given the individual variabilities seen in Fmr1 KO mice.

4. The MANOVA's are multiple comparisons, and multi-measure corrections are absent.

5. Although, the authors do justify the use of male mice in the methods, sex differences (especially in behavior) have been observed, and would should be acknowledged as a caveat.

Tissue collection: The authors mention the collection of several regions, but it's unclear why these regions were excluded from the results. Similarly, the rationale for mixing anatomically diverse regions, with variable cellular composition was not explained.

1. I am surprised the authors did not evaluate serum levels. That would have been informative and complemented their other measurements. Especially, since the authors seem to be directly extracting tissue (with no ACSF or saline perfusions which would normalize ionic balances across mice), tissue specific dysregulation could be a reflection of circulatory changes.

2. For clarity, consider re-ordering the order of sample collection. I am assuming the fecal collection was done first, followed by brain, cecal and fur.

3. I may have missed it, but there is no records of ages of mice from which samples were collected. It is unclear if ionic compositions are consistent across age and if this was assessed.

Figures

Figure 1- It would be clearer if the genotype were identified by symbols and tissue by color. That would give a good sense of the genotype differences.

Figure 2- Maybe the authors could change the symbol of the entries, or state that the black dots indicate outliers. Otherwise it gets confusing.

Confusing statements or references:

1. Line 13- 'comprehensive' is an overclaim. The study is a good 'proof of principle' of assessing ionomics as a viable and informative approach in FXS. A comprehensive study would require a lot more statistical power and precision in tissue extraction.

2. Line 28- typo. Salcedo-Arellano et al., is 2020 not 2023.

3. Line 40 This argument is confusing and logic inverted.

1. The references they use are bulk RNAseq and proteomic studies. These do not track single molecular pathways but capture system wide cellular perturbations. The ionome would reflect altered cellular processing (especially brain ionome), and hence would be a good complement but not be a substitute to transcriptomic or proteomic approaches.

2. Donnard 2022 is a single cell RNAseq study, not a bulk RNAseq study.

3. Missing references. For the bulk RNAseq Jung et al., 2023 PMID: 38048343 would support their results.

4. For proteomics Tang et al., PMID 26307763 should also be cited.

4. Line 45 Fmr1 should be italicized.

5. Line 49: Stefani 2004 does not link FMRP to identity of transcripts. HIT-clips studies Darnell 2011 and Maurin 2018 would be more appropriate references. Similarly Zhou et al., 2025 is not a neuronal paper, but a paper about the role of FMRP in tumor microenvironment.

6. Line 79; location of the reference (Greenacre 2021) is confusing as it seems to imply the reference refers to elemental reorganization in FXS. Maybe the placement was meant to be before?

7. Line 228: '... indicated ionomic similarity between genotypes, although PMHTH and striatum did not overlap', are the authors talking about genotype or tissue type comparisons?

8. Line 237 (Kooy, 2003) The authors are conflating human animal model face-validity with variability in rodent models. A better reference for the latter is Kat 2022 (PMID: 35690123)

9. Line 239, variance in 'both' not 'either'

10. Line 245: "FXS pathologies may also be expected ..." ionic dysregulation and neural excitability have been observed repeatedly in FXS, across several papers as highlighted in the previous line. Secondly, the references Napoli 2016 is about Zn, which is not affected in the brain. Similarly, D’Antoni et al., 2024, does not link FXS to trace elements or metalloenzymes. There are several links of FXS to metalloproteases and ECM (especially MMP9 among others). Please modify.

11. Line 268, The sentence is confusing and is unclear what the authors are trying to link. They refer to zinc-binding motifs (maybe proteins with zinc-binding motifs) and FMRP-pathways? Secondly, Edbauer 2010 is not an appropriate reference since as it does not link Zn and FXS).

12. Line 289, I disagree with the authors assertion of ionomics as a reverse genetics lens, to provide mechanistic insights, especially in complex multi-cellular tissue such as the brain. The papers the authors cite are papers from homogenous tissue or cell lines, where perturbing genes and linking to elements is possible. However, recent studies with cell specific manipulations (Fmrp knockouts, FMRP reexpression, or RNA translatome studies) suggest cell specific roles of FMRP (across neurons, interneurons and glia). Unless the authors are suggesting cell specific ionome (with cell-sorting), global ionome signals will be hard to resolve to the cellular source (genomic 'haystacks' are now cellular 'haystacks'). At best, the current iteration of ionome would be as a phenotypic tool and its applicability to tissue specific ionomic profiling in identifying tissue-element disruptions I agree. It might also be possible to link behavioral deficits to fecal elemental compostion, would potentially enable biomarker discovery (which the authors state).

13. Line 302, similar to my above comment, integrating global ionome with global transcriptome, proteome screens to identify cellular mechanisms, especially with cell specific dysregulation, is challenging.

**********

6. PLOS authors have the option to publish the peer review history of their article (what does this mean?). If published, this will include your full peer review and any attached files.

If you choose “no”, your identity will remain anonymous but your review may still be made public.

**Do you want your identity to be public for this peer review?**  For information about this choice, including consent withdrawal, please see our Privacy Policy.

Reviewer #1: **Yes:** Cara Westmark

Reviewer #2: No

**********

[NOTE: If reviewer comments were submitted as an attachment file, they will be attached to this email and accessible via the submission site. Please log into your account, locate the manuscript record, and check for the action link "View Attachments". If this link does not appear, there are no attachment files.]

To ensure your figures meet our technical requirements, please review our figure guidelines: https://journals.plos.org/plosone/s/figures

You may also use PLOS’s free figure tool, NAAS, to help you prepare publication quality figures: https://journals.plos.org/plosone/s/figures#loc-tools-for-figure-preparation.

NAAS will assess whether your figures meet our technical requirements by comparing each figure against our figure specifications.

# Author comment: Author response to Decision Letter 1

**DOI:** 10.1371/journal.pone.0352693.r002

6 Jun 2026

We thank the reviewers and editor for their suggestions for revision for our manuscript. We have made substantial changes including a new analysis pipeline that has significantly strengthened the manuscript. We have addressed reviewer’s concerns point by point in the document below.

Reviewer #1: This is a very interesting study measuring the multivariate balance of 10 elements in tissues of WT and Fmr1KO mice. The authors compared ion composition of gut, brain, fur and feces in WT and Fmr1KO mice. Differences are found as a function of brain region and genotype. Of note, higher iron is found in the olfactory bulb of Fmr1KO and higher magnesium and zinc in the feces of WT mice. The authors discuss the findings considering autism spectrum disorders. Overall, the study of ionomics is important, and relatively new, to the fragile X field potentially offering new biomarkers.

Thank you for the kind review and valuable feedback. We have incorporated suggestions and addressed concerns point by point below.

Issues to Address:

Abstract

Line 13: recommend changing “and ion flux” to “including mRNAs that code for proteins regulating ion flux”. - Updated

Lines 13-14: The statement, “a comprehensive comparison of elemental balance between FXS genotypes and tissues remains absent from the literature”, does not seem fair considering the work by Talvio et al measuring Na, Mg, P, K, Mn, Fe and Cu in cortex, cerebellum, liver, spleen and heart.

Updated to read

“However, there are few studies measuring the elemental balance between FXS genotypes and tissues.”

Introduction

Line 72: Since there is no global mechanism enforcing a fixed total elemental constant, why would elements be constrained to a constant sum? Aren’t elements regulated independently, i.e., sodium via kidneys and aldosterone; calcium via bone remodeling, PTH and vitamin D; and iron via absorption and recycling? In a related publication by Talvio and colleagues, they compared absolute amounts of elements in WT versus Fmr1KO mice.

We agree that biological systems do not possess a single physiological mechanism enforcing a fixed total elemental inventory across tissues. Individual elements are regulated through distinct physiological pathways operating at different rates across cells and tissues (e.g., renal handling of Na, hormonal regulation of Ca and P, iron absorption and recycling).

Our use of a compositional framework does not assume coordinated physiological regulation toward a biologically fixed elemental total. Rather, compositional analysis reflects the structure of multielement measurements themselves. Because tissues are open dynamical systems with simultaneous elemental uptake, storage, transport, utilization, and excretion occurring at different rates for different elements, interpretation of any single elemental abundance is necessarily contextualized by the relative abundances of other measured elements within the same sample. Compositional approaches are designed to account for these interdependencies and avoid artifacts associated with treating multielement measurements as unconstrained independent variables (Aitchison 1986; Greenacre et al. 2021). We revised the manuscript to clarify that our use of compositional data analysis arises from the relative nature of multielement measurements rather than from an assumption of a biologically fixed elemental total.

Methods

More detail should be added on animal husbandry. Were littermate mice used? What was the diet/bedding/etc. that may have affected mineral levels.

More information has been included on the husbandry and diet/bedding. See below:

“All mice were on a 12-hour light cycle (6 AM- 6 PM) and received the same chow (LabDiet 500, Lab Supply, Fort Worth, TX) 1 and bedding (alpha-dri, Innovive, San Diego, CA) and additional nestlets (cotton NST, Lab Supply, Fort Worth, TX).” We have also included information on husbandry including that all animals were littermates and therefore data reflect animals from two cages per genotype.

Discussion

Can the authors add to the discussion: (1) any limitations associated with the use of isoflurane that may affect mineral, particularly iron, levels?

We have added the following statement in our discussion section:

“Although isoflurane exposure was applied uniformly across experimental groups, previous studies have reported that inhalation of anesthetics can affect iron homeostasis, ferroptosis-related pathways, intracellular metabolite diffusion, and mineral-regulatory processes (Wang et al. 2019; Miao et al. 2024). Together, these findings suggest that isoflurane may affect elemental and metabolic homeostasis and therefore represents a potential confounding factor when interpreting tissue mineral composition data, particularly iron-related measurements, and is an important difference between our work and previous published literature (Talvio et al. 2021).”

(2) compare/contrast methods with Talvio such as CO2 versus isoflurane (see above). (3) Discuss the large difference in absolute mineral content between the current study and the Talvio study Table 3. Talvio has data for cortex and cerebellum, and the authors have PMHTH and olfactory brain. It looks like about a 10-fold difference for iron, and other elements also exhibit large differences. (4) Discuss future directions including the need to assess labile mineral content. The extent of iron toxicity depends on localization of the iron within the cell and its biochemical form.

We have added the following to the discussion:

(3) While previous work has also measured elemental differences in FXS, direct quantitative comparison between the present study and others should be interpreted cautiously because the studies differed substantially in tissue selection, analytical methodology, and normalization strategy. Talvio et al. quantified absolute elemental concentrations (µg/g wet weight) in cortex, cerebellum, heart, liver, and spleen using ICP-MS, whereas our study analyzed dried tissue samples using ICP-OES, which is less sensitive, within a compositional framework focused on relative multielement organization across olfactory bulb, striatum, PMHTH, fur, feces, and cecal contents. Additionally, the studies differed in tissue preparation and experimental workflow, including anesthetic exposure, tissue pooling strategy, drying procedures, digestion workflows, and elemental normalization approaches. These methodological differences are expected to influence absolute elemental concentrations, particularly for redox-sensitive elements such as iron. Importantly, Talvio et al. similarly emphasized cautious interpretation due to limited statistical power and multiple comparisons (Talvio et al. 2021). Our study was designed to evaluate whether loss of Fmr1 alters relative multielement organization across neural and somatic tissues. In this compositional framework, interpretation primarily depends on relative elemental relationships rather than direct equivalence of absolute concentrations.

(4) “Even though, in this study we measured overall relative concentration of an element in different neural as well as somatic tissues, future studies should assess not only total elemental abundance but also labile mineral content. The reason behind this logic is total tissue mineral levels do not unveil whether an element is biologically in active form, safely stored, protein-bound, redox-active, or localized within specific cellular compartments in tissues. This especially carries crucial insights for iron, because iron toxicity depends strongly on its biochemical form and intracellular localization. For example- ferritin-bound or transferrin-bound iron is relatively protected, whereas labile Fe²⁺ can promote Fenton chemistry, oxidative stress, lipid peroxidation, and ferroptosis (Kakhlon and Cabantchik, 2002). Thus, two tissues may show similar total iron concentrations, yet differ substantially in toxicity or physiological impact if one contains a larger labile/redox-active iron pool. Therefore, measuring only total tissue iron could not fully capture biologically relevant alterations in iron homeostasis in our study.

For the other elements measured in this study — their labile pools may provide more mechanistic insight than total elemental concentration alone. For example, labile Ca²⁺ regulates ion transport, secretion, epithelial signaling, and cellular stress responses (Berridge et al. 2003); labile Zn²⁺ functions as a signaling ion (De Leon-Rodriguez et al. 2012); and labile Cu and Mn may influence redox biology, mitochondrial function, and oxidative stress (Lindahl and Moore 2016).

Future studies should investigate labile metal pools within the gut lumen and microbial environment, because total elemental abundance in luminal contents may not accurately reflect the fraction of minerals that is biologically available to microbes or host epithelial cells. Since the concept of “Labile metals” refers to exchangeable, bioavailable metal ions that actively participate in microbial metabolism, redox reactions, signaling, and host–microbe interactions rather than metals that are tightly bound or inertly stored. Previous studies also emphasize that bacteria dynamically regulate labile metal pools to maintain metabolic homeostasis, oxidative stress resistance, and nutrient acquisition (Helmann 2025).

This is particularly relevant to our study because we quantified elemental concentrations in intestinal luminal contents, where metals are simultaneously influenced by diet, microbial utilization, epithelial transport, mucus interactions, and host inflammatory responses (Hood and Skaar 2012; Lopez and Skaar 2018). Therefore, future studies should examine not only total elemental levels but also the biologically active and compartment-specific metal pools, as these may better explain how altered mineral homeostasis contributes to gut microbiome changes and other neural as well as peripheral phenotypes in FXS.

Therefore, future work is needed to combine bulk elemental profiling with approaches that measure bioavailable and compartment-specific mineral pools. This would help determine whether altered mineral composition in FXS reflects changes in total abundance, altered intracellular distribution, or shifts in the biologically reactive mineral pools most likely to affect mitochondrial metabolism, oxidative stress and cellular toxicity.

(5) The olfactory bulb is one of the most iron sensitive regions of the brain. The authors could discuss how their results fit with the Fmr1KO mouse literature on olfactory bulb outcomes.

We subsequently shifted analytical strategies due to suggestions to include mixed models, and updated results do not reflect previously observed differences.

Reviewer #2: This study by Alam et al. carries out a comparison of elemental ion balance between male Fmr1 KO mice and WT mice of 3-5 months of age, sampling from several brain regions and somatic tissue. Given prior findings of ionic dysregulation in FXS literature, these results and approach to evaluate global ionomics is of interest to a broader FXS and ASD audience. The measurements across the brain regions and gut (cecal and fecal) is novel, the analytical framework useful and the potential for multi domain comparisons intriguing.

Given the novelty of assessing applicability of 'ionomic' analysis to ASD and FXS literature, I would support publication, if the following concerns are addressed.

Broadly, the major concerns are analytical discrepancies (which seem typographical errors) and unshared data which raise concerns. Moderate concerns are incorrect citations, toning down the conclusion of the writing (especially) in the discussion which should be more precise and rigorous.

Thank you for your insights and suggestions which we have incorporated below in a point-by-point response. We have since made sure that all of our data is now available – see updated statement with links to the data.

Major Concern 1)

1. Although, the data availability statement states that data is available and relevant data within the manuscript, I could not find – see updated statement with links to data

1) average element concentrations for each sample.

2) the ALR-transformed values.

3) details about both the missing values (which samples, tissue and genotype), and tables of imputed numbers. This is especially critical for Zn as there were 7 missing values and Zn was one of the elements that was significantly dysregulated in a genotype specific manner.

We corrected the number of missing Zn values to 8 and specified the sample information. Additionally, we have provided the raw data csv along with code to produce all analyses with these revisions, including initial imputation and alr-transformation.

The only available data set was the Mean SEM in Table S8 and S9 and here there a couple of glaring inconsistencies,

1. Table S9- large numbers. For example, striatum WT is 74.6 ± 0.006 µg/mg whereas KO is 0.067 ± 0.004 µg/mg. (Is this a typo? 0.074 would match their results). Similarly, fur calcium is transcribed as WT 39.2, KO 6.2. These numbers are unusually large.

2. Table S8, if derived from S9 (by normalizing to Fv), do not reflect these proportions.

The results in the table S8, for most parts match the conclusions. However, none of the mean difference findings reach significance when tested from Table S8 values (since the test were on ALR transformed values). Without the availability of ALR transformed values, which is missing, these results cannot be verified.

Erroneously large values were typos in previous tables - we thank the reviewer for their diligent and careful eye.

Methodological concerns.

Study design:

1. Please expand on the breeding strategy (Hom x Hom, Hom x WT) and housing information (were mice single or grouped housed). Gut microbiome would normalize if grouped housed and cage effects would need to be considered.

Our new comprehensive Bayesian multivariate mixed model approach should account for this variance as well. In our analysis the contribution of individual animal variation was low, and we expect this would be similar for group-housing effects. We have included more information in the methods on housing (group, etc. and breeding scheme). All our data were from group-housed individuals which results in only two cages per genotype.

2. Please state in the methods that all samples are from the same individual (if this is the case)

If samples are from within an individual mouse, it is unclear why the authors did not model this into their analysis. The MANOVA pools all observations together, but those observations are not truly independent, violating the independence assumption. A multivariate mixed model would have been more appropriate. If the authors did try this, and did not have sufficient power, this should be acknowledged in the results.

In response to this and the subsequent comment, we have wholly shifted our analytical framework to a Bayesian multivariate mixed model to allow better inference of variance partitioning.

3. The authors missed an opportunity to compare within individual, cross-tissue correlations (which would have been possible with a mixed model). This would have been a good comparison to have given the individual variabilities seen in Fmr1 KO mice.

4. The MANOVA's are multiple comparisons, and multi-measure corrections are absent.

MANOVA’s and PCA have subsequently been replaced with a Bayesian mixed-modeling framework which also addresses within individual correlations.

5. Although, the authors do justify the use of male mice in the methods, sex differences (especially in behavior) have been observed, and would should be acknowledged as a caveat.

We added this to the discussion:

“Additionally we only used male mice in our study, previous work has shown that there are sex differences physiologically (Chawla and McCullagh 2022; Smith et al. 2021) and behaviorally (Petroni et al. 2022) in FXS mice suggesting that sex may also be an important factor for future work.

Tissue collection: The authors mention the collection of several regions, but it's unclear why these regions were excluded from the results. Similarly, the rationale for mixing anatomically diverse regions, with variable cellular composition was not explained.

We

Attachment Submitted filename: Ionome PLoS one_reviewer.docx

# Peer review: Decision Letter 1

**DOI:** 10.1371/journal.pone.0352693.r003

**Authors:** Nien-Pei Tsai

15 Jun 2026

Loss of Fmr1 reorganizes the multi-elemental composition across tissues in Fragile X Syndrome mice

PONE-D-26-15087R1

Dear Dr. McCullagh,

We’re pleased to inform you that your manuscript has been judged scientifically suitable for publication and will be formally accepted for publication once it meets all outstanding technical requirements.

Within one week, you’ll receive an e-mail detailing the required amendments. When these have been addressed, you’ll receive a formal acceptance letter and your manuscript will be scheduled for publication.

An invoice will be generated when your article is formally accepted. Please note, if your institution has a publishing partnership with PLOS and your article meets the relevant criteria, all or part of your publication costs will be covered. Please make sure your user information is up-to-date by logging into Editorial Manager at Editorial Manager® and clicking the ‘Update My Information' link at the top of the page. For questions related to billing, please contact billing support.

If your institution or institutions have a press office, please notify them about your upcoming paper to help maximize its impact. If they’ll be preparing press materials, please inform our press team as soon as possible -- no later than 48 hours after receiving the formal acceptance. Your manuscript will remain under strict press embargo until 2 pm Eastern Time on the date of publication. For more information, please contact onepress@plos.org.

Kind regards,

Nien-Pei Tsai, PhD

Academic Editor

PLOS One

Additional Editor Comments (optional):

Reviewers' comments:

Reviewer's Responses to Questions

**Comments to the Author**

1. If the authors have adequately addressed your comments raised in a previous round of review and you feel that this manuscript is now acceptable for publication, you may indicate that here to bypass the “Comments to the Author” section, enter your conflict of interest statement in the “Confidential to Editor” section, and submit your "Accept" recommendation.

Reviewer #2: All comments have been addressed

**********

2. Is the manuscript technically sound, and do the data support the conclusions?

The manuscript must describe a technically sound piece of scientific research with data that supports the conclusions. Experiments must have been conducted rigorously, with appropriate controls, replication, and sample sizes. The conclusions must be drawn appropriately based on the data presented.

Reviewer #2: Yes

**********

3. Has the statistical analysis been performed appropriately and rigorously?

Reviewer #2: Yes

**********

4. Have the authors made all data underlying the findings in their manuscript fully available?

The PLOS Data policy requires authors to make all data underlying the findings described in their manuscript fully available without restriction, with rare exception (please refer to the Data Availability Statement in the manuscript PDF file). The data should be provided as part of the manuscript or its supporting information, or deposited to a public repository. For example, in addition to summary statistics, the data points behind means, medians and variance measures should be available. If there are restrictions on publicly sharing data—e.g. participant privacy or use of data from a third party—those must be specified.

Reviewer #2: Yes

**********

5. Is the manuscript presented in an intelligible fashion and written in standard English?

PLOS ONE does not copyedit accepted manuscripts, so the language in submitted articles must be clear, correct, and unambiguous. Any typographical or grammatical errors should be corrected at revision, so please note any specific errors here.

Reviewer #2: Yes

**********

6. Review Comments to the Author

Please use the space provided to explain your answers to the questions above. You may also include additional comments for the author, including concerns about dual publication, research ethics, or publication ethics. (Please upload your review as an attachment if it exceeds 20,000 characters)

Reviewer #2: The authors have addressed all concerns admirably. Their analysis is rigorous, transparent and interpretation appropriate. I am happy to support publication.

**********

7. PLOS authors have the option to publish the peer review history of their article (what does this mean?). If published, this will include your full peer review and any attached files.

If you choose “no”, your identity will remain anonymous but your review may still be made public.

**Do you want your identity to be public for this peer review?**  For information about this choice, including consent withdrawal, please see our Privacy Policy.

Reviewer #2: No

**********

# Editor report: Acceptance letter

**DOI:** 10.1371/journal.pone.0352693.r004

**Authors:** Nien-Pei Tsai

PONE-D-26-15087R1

PLOS One

Dear Dr. McCullagh,

I'm pleased to inform you that your manuscript has been deemed suitable for publication in PLOS One. Congratulations! Your manuscript is now being handed over to our production team.

At this stage, our production department will prepare your paper for publication. This includes ensuring the following:

* All references, tables, and figures are properly cited

* All relevant supporting information is included in the manuscript submission,

* There are no issues that prevent the paper from being properly typeset

You will receive further instructions from the production team, including instructions on how to review your proof when it is ready. Please keep in mind that we are working through a large volume of accepted articles, so please give us a few days to review your paper and let you know the next and final steps.

Lastly, if your institution or institutions have a press office, please let them know about your upcoming paper now to help maximize its impact. If they'll be preparing press materials, please inform our press team within the next 48 hours. Your manuscript will remain under strict press embargo until 2 pm Eastern Time on the date of publication. For more information, please contact onepress@plos.org.

You will receive an invoice from PLOS for your publication fee after your manuscript has reached the completed accept phase. If you receive an email requesting payment before acceptance or for any other service, this may be a phishing scheme. Learn how to identify phishing emails and protect your accounts at https://explore.plos.org/phishing.

If we can help with anything else, please email us at customercare@plos.org.

Thank you for submitting your work to PLOS ONE and supporting open access.

Kind regards,

PLOS ONE Editorial Office Staff

on behalf of

Dr. Nien-Pei Tsai

Academic Editor

PLOS One
